# Estimation of the marginal effect of regular drug use on multiple sclerosis in the Iranian population

**DOI:** 10.1371/journal.pone.0196244

**Published:** 2018-04-24

**Authors:** Ibrahim Abdollahpour, Saharnaz Nedjat, Mohammad Ali Mansournia, Tibor Schuster

**Affiliations:** 1 Department of Epidemiology, School of Public Health, Arak University of Medical Sciences, Arak, Iran; 2 Department of Epidemiology and Biostatistics, School of Public Health, Tehran University of Medical Sciences, Knowledge Utilization Research Center, Tehran University of Medical Sciences, Tehran, Iran; 3 Department of Epidemiology and Biostatistics, School of Public Health, Tehran University of Medical Sciences, Tehran, Iran; 4 Department of Family Medicine, McGill University, Montréal, Quebec, Canada; Karolinska Institutet, SWEDEN

## Abstract

There are only few reports regarding the role of lifetime drug or substance use in multiple sclerosis (MS) etiology. In this study, we investigated the potential effect of drug or substance exposure on the onset of MS diagnosis. We conducted a population-based incident case control study in Tehran. Cases (n = 547) were 15–50 years old persons with MS identified from the Iranian Multiple Sclerosis Society (IMSS) register during August 7, 2013, and November 17, 2015. Population-based controls (n = 1057) were 15–50 years old and were recruited by random digit telephone dialing. Inverse-probability-of-treatment weighing (IPTW) using two sets of propensity scores (PSs) was used to estimate marginal incidence odds ratios (ORs) for MS contrasting pre-specified substance use. The estimated marginal OR was 6.03 (95% confidence interval: 3.54;10.3, using trimmed weights at the 95^th^ percentile of the stabilized weight distribution) in both IPTW analyses comparing lifetime substance use (opioids, cannabis, inhalants, hallucinogens and stimulants) for at least one time monthly during a six-months or longer period vs. no such history of drug use. Subject to limitation of causal claims based on case-control studies, this study suggests that monthly drug or substance use for a period of at least six consecutive months, may increase the risk of MS by factor 3.5 or higher.

## Introduction

Multiple sclerosis (MS) is a leading cause of non-traumatic disability in young adults. The estimated global number of persons living with MS has increased from 2.1 million in 2008 to 2.3 million in 2013[1]. Recently, the observed critical rise in the incidence of MS, has introduced Tehran as a high-risk area for MS[2]. Although the etiology of MS remains poorly understood, there is evidence suggesting potential roles of both environmental and genetic factors in the development of MS. Nonetheless, the effects of (possibly different definitions of) drug or substance use, as modifiable potential risk factors on MS onset have not been fully studied. Due to the relatively low prevalence of MS in target populations, case-control studies are commonly utilized to estimate associations of drug or substance use and MS.

In situations where comprehensive data on known or suspected confounding variables are collected (and under further regularity assumptions), advanced causal inference methods can be applied to estimate average exposure effects that reflect contrasts in population-level outcomes under counterfactual population-wide exposure scenarios [3–7]. For example, inverse-probability-of-treatment weighting (IPTW) using the propensity score (PS)[6] and model-based standardization [3,4] have been previously used for estimating the average causal effects based on case-control studies. Although applications of the PS in observational studies, as a balancing score, had been primarily restricted to cohort studies, its estimation and application in case-control studies has been discussed more recently[6]. In fact, it is possible to consistently estimate the PS in case-control studies using (i) controls, or (ii) the total sample when applying weights that correspond to the inverse of case and control sampling fractions[6]. While valid estimation of the propensity score using method (ii) requires the knowledge of the sampling fraction of the study groups, PS estimation employing method (i) is approximately correct under the rare disease assumption. The aim of this study was to estimate the average causal effect of monthly drug or substance use for a period of at least 6 consecutive months on MS in the Iranian population. We used inverse-probability-of-treatment weighting (IPTW) based on the subpopulation of controls as well as in the setting of a population-based incident case-control study with known sampling fractions for both cases and controls.

## Materials and methods

### Participants

We conducted a population-based incident case-control study with all residents of 22 municipality areas of Tehran aged 15 to 50 years between August 7, 2013, and November 17, 2015[8]. The primary study base therefore included nearly 5.11 million persons. Incident cases were identified using the Iranian Multiple Sclerosis Society (IMSS), as the only registry in Tehran. For estimating the completeness of case recruitment via IMSS, we calculated the expected number of incident cases during the study period. Based on the latest report in Tehran in 2013, the age-adjusted incidence rate was 6.6 per 100000 per year [9]. Accordingly, with a population of 5.11 million observed over 1.5 years, the occurrence of 506 new cases (337 new cases per year) is expected. The actual number of incident cases retrieved from the IMSS case database was 570. All incident patients in the IMSS register had a clinical diagnosis of MS from at least one neurologist using the 2010 McDonald criteria [10] and were required to have a confirmed diagnosis based on medical imaging i.e. magnetic resonance imaging. The index date for cases was defined as the year and month in which individuals from the IMSS register received their confirmed diagnosis. All identifiable cases in the database were included in the study sample. Using random digit dialing (RDD), 1057 general population controls aged 15 to 50 years who were, at the time of case diagnosis, alive and in the study base participated in the study. We selected controls proportional to the sizes of the 22 considered municipality areas of Tehran. In the case of nonresponse, another control was selected using the same protocol. The sampling fraction for controls was approximately 2:10000 (1057/5115679). The study was approved by the “ethics in research committee” of Tehran University of Medical Sciences with the approval number 26145.

#### Random digit dialing protocol

We used standard method of random digit dialing (RDD) for identifying eligible controls [11]. For this, the existent pre-codes of 22 municipality areas were completed by four randomly generated digits. If the complete number was linked to an active residential phone, the number was included on the study interview calling list; otherwise, the number was discarded. The similarity of RDD selected controls with address-based sampling (ABS) controls [12], its usefulness, effectiveness and feasibility [11,13,14] has been formerly demonstrated. Based on our RDD protocol, to identify and select potential controls, a maximum of nine calls (two times in the morning, two times in the afternoon or evening and five times at different times on other days) were made before a random digit could be dropped. A screening interview, to determine whether any household member fulfilled the study entry criteria, was initially conduced. To match with the age distribution of cases, we considered only 15–50 years old residents as eligible for the study. The screening was also used to identify potential MS patients that were not registered as cases by assessing history and presence of clinical signs and symptoms of MS. We applied the Kish method for random selection of eligible members of each selected household [15,16].

In order to retrieve exposure and covariate information from cases, all identified persons with confirmed MS diagnosis from the IMSS register were called using the same protocol as for controls (maximum of nine calls: two times in the morning, two times in the afternoon or evening and five times at different times on other days).

#### Data collection

The phone interviews were conducted by 10 specially trained interviewers to ensure a standardized interview and data collection process. For this purpose, all interviewers had to follow the same detailed protocol. The data collection activities of interviewers were monitored to enable detection of possible interviewer bias. For example, interviewers were not supposed to provide unnecessary interpretation of survey questions that could possibly affect the response of the interviewed person. Also, at the start of each interview, the main objectives of study were clearly elucidated for all of study participants. To minimize potential selection and/or response bias, potential participants were informed that all information provided will be processed anonymously and that no legal consequences could arise regarding their responses. Finally, the following information was equally retrieved from cases and controls using phone interviews: demographic factors as well as lifestyle variables and history or presence of depression. In the beginning of each interview, the study main objectives were enumerated and informed verbal consent was obtained from all participants. In the case that participants were less than 18 years old, the informed verbal consent was obtained from their parents.

#### Exposure

Any recent or past drug/substance use was extracted via the following question: *“Have you ever used any type of substance (Opioids*, *Cannabis*, *Inhalants, Hallucinogens and Stimulants) for at least one time monthly during a period of 6 month or longer?”* Detailed information on its type (opioids, cannabis, inhalants, hallucinogens and stimulants), duration (years), amount (average frequency per month) was also obtained. The primary study exposure was defined as self-reported lifetime history of any monthly drug use that occurred for a period of at least six months. For cases, this period had to be entirely located before the index date (time of diagnosis) and for controls before the respective interview date.

### Statistical methods

#### Pre-specified confounding variables

The participants were requested to provide all information for the time before index date if they were cases and similarly, before the sampling date, if they were controls. Total lifetime ethanol intake was calculated through the University of Minnesota's nutrient data system [17,18]. Passive smoking was assessed by asking study participants the following question: *“Have you ever lived in a home for at least 12 months with one regularly smoker during your lifetime?”* Participants were requested to rate their family SES using the following question: *“How do you rate your family socio economic status on a scale from 1 (low) to 10 (high)?”*[19]. We used current knowledge based on evidence from literature for determining which covariates are risk factors for MS. A causal diagram [20,21] representing the effect of drug or substance use on MS was used to identify the minimal sufficient set of covariates to be controlled for in the inference models. We excluded covariates associated with the drug use but that were assumed to have only little or no impact on MS from the exposure model for efficiency and positivity reasons [22]. We additionally included variables which are unlikely to be related to drug or substance use but strongly associated with MS (e.g., passive smoking [8,23]), because this likely increases the precision of the effect estimate without increasing bias [24]. Based on existing literature the following covariates were selected as potential confounders for the effect of drug or substance use on the incidence of MS: socio-demographic and life style factors including age, sex, highest level of education (Illiterate or primordial, guidance, high school, associate's or bachelor degree and master's degree and higher), cigarette pack-year (never, ≤5, > 5 pack-year), passive smoking history (yes / no), history of smoking during adolescence (yes / no), cumulative amount of water pipe smoking, as well as social economic status (SES). Non-categorical confounding variables such as age, alcohol intake and social economic status were flexibly modeled using cubic b-spline functions in the respective propensity score models.

#### Inverse-probability-of-treatment weighting

To address potential confounding bias that could be introduced by group imbalances with respect to measured covariates, inverse probability of treatment weighting (IPTW) was used when estimating incidence odds ratios (ORs).

Two sets of propensity scores (PSs) were estimated by fitting two binary logistic regression models with the previously defined drug or substance use criterion as response variable and pre-specified confounders as predictor variables among (i) controls, and (ii) the total sample (cases and controls). When estimating the propensity scores for the total sample, sampling fractions were used as weights to emulate representativeness of the sample data i.e. 1 for cases and 1/(1057/ 5115679) = 4839.81 for controls [6]. Based on the estimated propensity scores, stabilized weights were calculated dividing the prevalence of the exposure received (drug or substance use according to the previously specified definition) by the estimated propensity score to receive the respective exposure. We note that, due to the case-control design of the study, prevalence values in the numerator of the stabilized weights were estimates based on the control population. We then estimated the marginal causal effect of drug or substance use by employing a weighted logistic regression analysis with MS status as binary response variable, past drug or substance use as single covariate and the stabilized inverse probability weights as weighting factor.

Validity of the inverse-probability-of-treatment weighted estimates using method (i) and (ii) rely on assumptions of no unmeasured confounding, positivity, consistency and correct specification of the exposure (drug or substance use) model. Method (i) further requires the rare disease assumption which holds for MS (its prevalence in Tehran approximately ranges from 5.3 to 74.28/100,000) [2].

## Results

### Response rates

During the RDD process, approximately 36.6% of the generated random digits revealed to be residential phone numbers of which 1591 were finally reached. We were able to retrieve data on medication or substance use and potential confounding variables from 1057 (70%) of the interviewed persons. The response rate among the 570 incident cases was 96%, yielding to 547 case data series on medication or substance use and potential confounding variables. Table 1 shows that there was a higher proportion of females (73.3%) among the cases compared to the controls (51.5%). The mean age was slightly lower for cases than controls and the current socioeconomic status was a little lower in cases than controls (Table 1).

**Table 1:** Characteristics of MS cases and controls, Tehran, 2013–2015.

Variables	MS cases N (%)	Controls N (%)
**Gender**		
Female	401 (73.3)	544 (51.5)
**Age; mean (SD)**	31.30 (9.3)	30.50 (7.5)
**SES (1–10); mean (SD)**	5.25 (1.9)	5.40 (2)
**Marriage status**		
Single	213 (39)	462 (43.7)
Married	300 (54.9)	567 (53.6)
Single due to death/ divorce	34 (1.05)	24 (2.30)
**Highest level of education**		
Illiterate or primordial	10 (1.83)	26 (2.46)
Guidance	27 (4.94)	60 (5.68)
High school	197 (36.01)	437 (41.34)
Associate's or bachelor degree	255 (46.62)	441 (41.72)
Master's degree and higher	58 (10.60)	93 (8.80)
**Cigarette smoking–cumulative amount (total pack-years)**		
Never	438 (80.22)	843 (79.75)
≤ 5	79 (14.50)	165 (15.60)
> 5	29 (5.30)	46 (4.40)
**Passive smoking**		
Never	261 (47.80)	664 (63.00)
Ever	285 (52.2)	390 (37.00)
**Waterpipe smoking–cumulative amount**		
Never	380 (69.60)	803 (76.40)
≤ 250	85 (15.40)	121 (11.5)
> 250	81 (15.00)	127 (12.1)
**Lifetime alcohol consumption (gr)**		
Never	394 (72.83)	805 (77.55)
≤ 500	42 (7.76)	47 (4.53)
500–5000	55 (10.17)	76 (7.32)
> 5000	50 (9.24)	110 (10.60)
**Lifetime drug use**		
Yes	62 (11.4)	70 (6.6)

### Inverse probability weighting

**Fig 1:**
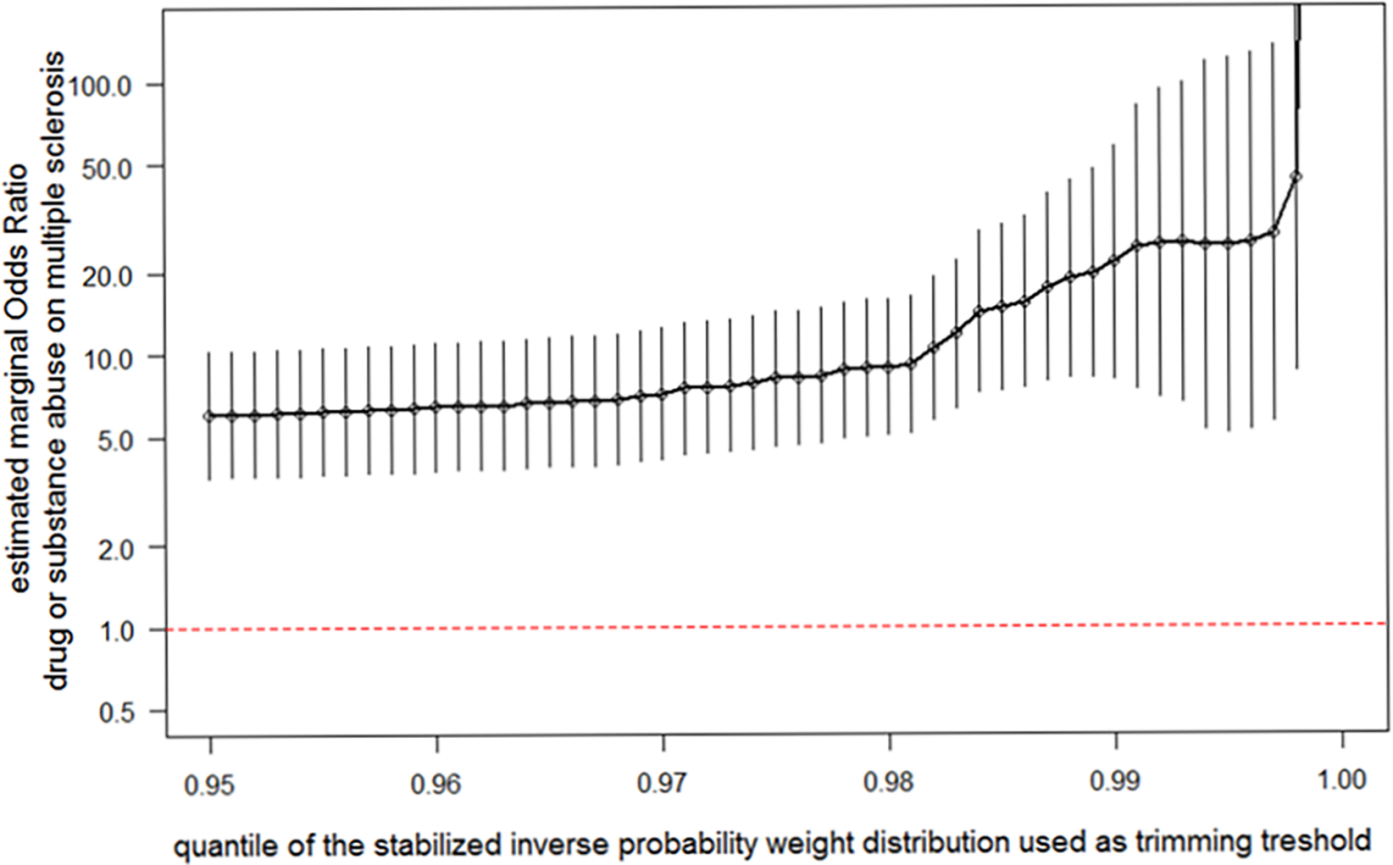
Estimated marginal odds ratios with 95% confidence intervals after applying different thresholds for weight trimming ranging from the 95^th^ to the 99.9^th^ distribution quantile of the stabilized weights.

Both exposure models, i) based on controls only and ii) based on cases and controls employing sampling weights, revealed almost identical distributions of estimated propensity scores. This concordance is explained by the very low effective weight cases received in the analysis. Despite stabilization of the weights from the two propensity score models, both weight distributions entailed a number of extreme values (weighting factors > 1,000,000) that were likely to distort the overall estimates of marginal exposure effects. We therefore employed trimming of the weight distribution which has been shown to improve the accuracy and precision of parameter estimates [25]. Fig 1 displays the estimated marginal odds ratios with 95% confidence intervals after applying different thresholds for weight trimming ranging from the 95th to the 99.9th distribution percentile of the stabilized weights. Based on this analysis, the lowest (most conservative) estimate for the effect of regular drug or substance use (according to the pre-specified definition) on multiple sclerosis, is a marginal odds ratio of 6.03 (95%CI: 3.54 to 10.3). Given the identity of the two propensity score distributions, this estimated odds ratios and confidence intervals were the same using either propensity score model.

## Discussion

In this large population-based incident case-control study, we identified lifetime drug use for at least one time monthly during a period of at least 6 months as a considerable risk factor for MS. According to the statistical analysis, using inverse probability weighting based on controls and the entire study sample, the chosen exposure definition is suggested to increase the incidence odds of MS for six times compared to individuals who do not meet the exposure definition (95% confidence interval 3.5 to 10.3).

This finding is consistent with the results of another case-control study conducted on sex and age-matched rheumatoid arthritis cases and controls which demonstrated drug use as a risk factor for MS [26]. Hawkes et al. conducted a case-control study that compared 94 clinically definite MS cases with 59 controls who were patients with benign headache. The findings of this study provided supporting evidence for an association of recreational drug use and MS 3.90 (95% CI: 1.32–11.50; p = 0.014) [27]. One potential mechanism underlying this detected effect of drug use on MS is a possible damage to the central nervous system e.g. atrophy in brain stem or cerebrovascular accidents problems, dependent on the type of consumed drug or substance [26]. However, there is also evidence in literature suggesting a weaker or even absent link between drug use and the onset of MS (OR = 0.91 (0.84–1.54)) [28]. These incompatible estimates can be partially explained by differences in the underlying study populations and deviating exposure and outcome definitions. Furthermore, the unknown nature of MS etiology may play an important role. In other words, there are potentially different sets of sufficient causes for MS. The estimated effect of drug use on the MS may largely dependent on the prevalence of specific factors comprising one distinct sufficient cause. Moreover, the lack of relevant information regarding the induction time of drug in MS development may be another potential source for this controversy.

The validity of the reported OR estimates using IPW and depends on the underlying assumptions including no residual confounding and correct model specification. The validity of IP-weighted ORs also requires no violation of the positivity assumption. We included known risk factors associated with drug use in study controls in our exposure and outcome models. To avoid inefficiency and non-positivity problems, we did not select variables that were only associated with drug use in study controls but were not risk factors for MS[29].

Our results should be interpreted with some limitations in mind. With all causal analyses including those used in this paper, it can only be expected that the effects of measured, but not unknown or unmeasured confounders are removed. Therefore, unmeasured and unknown covariates can still be imbalanced between drug users and non-users leading to residual confounding. Moreover, the possibility of measurement error in study main exposure as well as the other covariates due to recall bias or reporting bias should not be ignored. Measurement error in self-reported confounders can lead to residual confounding. The direction of the bias due to measurement errors in exposure and confounders is unpredictable without knowledge of error structures. The possibility of selection bias is another issue threatening the validity of the findings of this study. However, at least for cases we were able to retrieve a complete sample and a high response rate of 96%. On the other hand, using RDD for selecting population-based controls can alleviate the possibility of selection bias. Although, the response rate in control group was significantly smaller than cases, compared with other studies, this may be considered as a relatively high response rate [30]. Our estimated lifetime prevalence of drug use is moderately higher than one estimated by Amin-Esmaeili et al. in Iranian household Mental Health Survey (IranMHS) in which the 12-month prevalence of illicit drug use was slightly more than 2.1%, 95% CI: 1.70–2.47%. These findings suggest that the probability of underreporting drug or substance use in our study is at least less than in the IranMHS[31].

In conclusion, based on our findings, lifetime monthly drug use for a period of at least 6 months, as a modifiable risk factor, is possibly linked with an increased risk of MS.

## Supporting information

S1 Data (ZIP)

## Abbreviations

IMSS: Iranian Multiple Sclerosis Society
RDD: Random digit dialing
